# Support needs for chronic disease self-management among persons attending a Primary Health Care facility in Victor Khanye, Mpumalanga

**DOI:** 10.4102/safp.v68i1.6264

**Published:** 2026-07-27

**Authors:** Wonder Hlongwane, Talitha Crowley

**Affiliations:** 1 School of Nursing, Faculty of Community and Health Sciences, University of the Western Cape, Cape Town, South Africa

**Keywords:** chronic disease, diabetes, hypertension, HIV, self-management

## Abstract

**Background:**

With the increasing burden of non-communicable chronic diseases, it is important to empower people to self-manage their conditions. This study aimed to explore the support that persons living with chronic disease require to self-manage their conditions at a Primary Health Care (PHC) facility in the Victor-Khanye subdistrict, Delmas, Mpumalanga.

**Methods:**

Individual in-depth interviews were conducted with 14 adult participants who collected their monthly medication for diabetes, human immunodeficiency virus or hypertension. Data were analysed manually using thematic analysis.

**Results:**

Three themes were identified: (1) individual self-management; (2) support enhancing for self-management; and (3) barriers to self-management. The study highlights that self-management requires a combination of individual beliefs, capabilities, education, access to healthcare resources, financial assistance, emotional support from family and friends, and community support. While knowledge is crucial for empowering individuals to manage their diseases effectively, challenges such as financial constraints and a lack of specific self-management support provided by healthcare workers hinder many from fully implementing self-management strategies.

**Conclusion:**

Persons living with chronic disease require comprehensive care that extends beyond healthcare and includes emotional, social and financial support. Effective self-management requires access to tailored education and community support networks that allow people to take an active role in their health.

**Contribution:**

Understanding the support persons living with chronic diseases require for effective self-management is critical for improving health outcomes, enhancing quality of life and promoting sustainable healthcare systems.

## Introduction

The high burden of chronic communicable diseases, such as human immunodeficiency virus (HIV) infection, and the escalating rise of non-communicable diseases (NCDs) in South Africa and other sub-Saharan African countries, calls for a shift in how healthcare services are designed and delivered.^1^ The shift is towards empowering people to take greater responsibility for their health and to manage their chronic disease.^2^ Chronic diseases tend to be of a longer duration and result from an interplay of genetic, physiological, environmental and behavioural risk factors.^2^

To provide better care for individuals with chronic comorbid diseases, the South African Department of Health (DoH) implemented the Integrated Chronic Disease Management (ICDM) model in 2011 for Primary Health Care (PHC).^2^ The chronic diseases included in the ICDM are hypertension, HIV, diabetes, asthma, epilepsy, chronic obstructive pulmonary disease (COPD), tuberculosis and mental health illness.^2^ The ICDM model focuses on enhancing healthcare service delivery through facility reorganisation, clinical support, supported self-management and community-based support systems to provide efficient, patient-centred care.^2^ The overall aim of the ICDM is to provide integrated prevention, treatment and care for persons living with chronic diseases at the PHC level, to ensure the transition to assisted self-management within the community and achieve good outcomes for patients using the health system building blocks.^2^ These building blocks include service delivery, health workforce, health information systems, access to critical medications and technological resources, health financing, and leadership and governance.^3^ The ICDM model further recognises that most persons (70% – 80%) with chronic diseases can be managed in the community through self-management support.^4^

Self-management is described as a process by which individuals and families of persons living with chronic disease use knowledge and beliefs, self-regulation skills and abilities and social facilitation to improve their health outcomes.^5^ It takes place in the context of risk and protective factors specific to the condition, the physical and social environment, and the individual and family.^5^ Self-management for people with chronic diseases refers to the ability to manage their own symptoms, treatment, physical and psychosocial effects, and lifestyle adjustments that come with having a long-term disease.^4^ It includes responsibilities such as symptom monitoring, medication adherence, implementing essential lifestyle adjustments (such as eating healthy food, frequent physical activity and smoking cessation) and dealing with the emotional impact of chronic illness. For example, a person with diabetes may check their blood sugar levels regularly, adjust their diet, and take insulin as prescribed, whereas an asthmatic may avoid known triggers and use an inhaler correctly during flare-ups.^1^

Support for self-management plays a key role in achieving good health for persons living with chronic diseases and is an effective approach for long-term diseases.^5^ Mpumalanga is a small province located in the north-eastern part of South Africa. The major chronic diseases in the province are HIV, hypertension and diabetes.^6^ The province has initiated the ICDM and Centralised Chronic Medicine Dispensing and Distribution (CCMDD) strategies that assist in the management of persons living with chronic disease.^6^ The CCMDD is a programme that enables stable patients living with chronic diseases to access their medication more conveniently without needing to visit the clinic regularly. Although the ICDM has been implemented in several facilities in Mpumalanga and utilised by healthcare workers, the authors could not identify studies exploring the self-management support needs of persons living with chronic diseases conducted in this context.^4^ A systematic review found that contextual factors, such as socioeconomic status, may moderate the relationship between self-management support and health outcomes.^7^ This means that self-management support should be tailored to the context of individuals, families and communities.^7^ The present study, therefore, endeavours to explore the support that persons living with chronic disease require to self-manage their conditions at a PHC facility in the Victor-Khanye sub-district, Mpumalanga. This may assist in tailoring self-management support for persons living with chronic diseases in rural PHC contexts.

## Research methods and design

### Study design

A qualitative exploratory-descriptive approach was used in this study. This study was exploratory, as it examined knowledge of the subject under investigation.^8^ The researcher aimed to explore and describe the understanding and beliefs, self-management practices, and support of persons living with chronic disease regarding their disease through in-depth interviews.^9^ Exploratory research is particularly useful for determining the full nature of a phenomenon that is poorly understood.^10^ A qualitative exploratory design makes it possible for the study’s participants to contribute to the advancement of new knowledge in a subject that has received little attention.^5^

A descriptive research design was used; this design is used in research to describe or characterise a population, phenomenon or situation.^7^ This type of research aims to provide a comprehensive overview of the subject being studied without seeking to establish causal relationships or test hypotheses.^10^ Instead, it focuses on describing the characteristics, behaviours, attitudes or experiences of a group or phenomenon.^7^

### Study setting

The study was conducted at a PHC facility in the Victor Khanye sub-district, Delmas, Mpumalanga. Delmas is a semi-rural area located approximately 45 km from Johannesburg and includes four clinics serving township and farming communities. The area has an estimated population of 90 000–100 000 people. Although the population is relatively young, there is a growing number of adults and older persons vulnerable to chronic conditions such as hypertension, diabetes and multimorbidity, increasing the demand for chronic care services.^11^

The area experiences moderate to high unemployment, with many households relying on social grants, informal employment or low-income agricultural work. These socioeconomic challenges may affect access to transport, healthy lifestyle practices and adherence to long-term.^11^ One of the facilities, Botleng Extension 3 clinic, was selected based on accessibility and the number of people accessing chronic medication in the facility. The clinic serves more than 200 patients daily and a range of chronic diseases, including HIV, hypertension and diabetes.

### Study population and sampling

Approximately 4000 patients living with chronic conditions receive medication at the healthcare facility every month. Purposive sampling was used to select participants. To ensure maximum variability, the sample included participants with various chronic diseases, hypertension, diabetes and HIV with different languages, ages and genders.^8^ The inclusion criteria included males and females, adults living with chronic diseases (hypertension, HIV, or diabetes) attending the selected facility, and who have been taking chronic medication for at least 6 months.

The exclusion criteria were participants who could not speak English, Isizulu or Xitsonga because the researcher is only fluent in the mentioned languages.

The recruitment process began by introducing the study to the patients in the facility hall in the morning, before they were allocated to the consultation rooms. Twenty patients initially agreed to participate; however, only 14 attended the interviews. Data saturation was reached after 12 interviews and confirmed with two additional interviews.

### Data collection method and instruments

Individual in-depth interviews were conducted using a semi-structured interview guide between March 2023 and January 2024. The interview consisted of open-ended questions to allow the participants to express themselves. The interview guide was structured according to the individual and family self-management theory, which identifies key self-management processes, including knowledge and beliefs, self-regulation skills and abilities and social support resources.^12^ The theory was used as a framework to explore the support needs of patients living with chronic diseases (Appendix 1). The full interview guide can be viewed in Appendix 2.

The participants and the first author agreed on the time and place where individual interviews took place, which was the facility. IsiZulu and Xitsonga were used to conduct the interviews. The interviews took between 30 and 45 min and were audio recorded.

### Data analysis

The audio recordings were transcribed verbatim. It was translated from isiZulu and Xitsonga to English by the first author. The first author analysed the data with the assistance of the co-author using Braun and Clarke’s six-step thematic analysis approach. Beginning with immersion in the data, initial codes were generated and organised into broader themes.^12^ The co-author co-coded the first few transcripts, and both authors reached consensus on the final coding framework. The themes were then reviewed collaboratively to ensure that they accurately reflected participants’ narratives, after which they were clearly defined and named. Finally, the findings were presented to illustrate key patterns related to the self-management support needs of persons living with chronic diseases.

### Trustworthiness

The first author lives in the neighbourhood where the study was carried out and could relate to the cultural practices and background of the participants. The researcher reflected on potential bias because of her familiarity with the community and used reflexivity to reduce its influence on data collection and analysis. To ensure trustworthiness, the first author kept a diary to write field notes during the interviews, summarised and asked clarification questions during the interviews to ensure a clear understanding of the participants’ responses. Data collection continued until no additional information emerged. The researcher employed peer debriefing with the co-author. During the interviews, the first author summarised and asked clarifying questions to ensure a clear understanding of each participant’s responses. The data collection and analysis processes and decisions were documented to support dependability and confirmability.

### Ethical considerations

The study was approved by the Mpumalanga Department of Health (reference: MP_202208_004) and by the University of the Western Cape’s Biomedical Research Ethics and Research Committee (BMREC) with ethical clearance number BM22/6/26. Written and signed informed consent was obtained from all the participants. Confidentiality was ensured through the secure storage of recordings, anonymisation of participants using identification numbers instead of names, and restricted access to the data.

## Results

### Participant characteristics

The researcher interviewed 14 participants (*n* = 9 men, *n* = 5 women). The ages ranged from 22–59 years. Four participants were living with HIV (*n* = 3 women, *n* = 1 man), five participants were living with hypertension (*n* = 4 men, *n* = 1 woman), four participants with diabetes (*n* = 3 men, *n* = 1 woman) and one participant with comorbid diabetes and hypertension. The years since diagnosis ranged from 1–22 years, with six participants out of the 14 participants under the age of 35 years (see Table 1). The inclusion of younger participants may reflect the growing burden of chronic diseases among younger adults in South Africa. The predominance of male participants may reflect the characteristics of patients attending the facility during the recruitment period and those willing to participate in the study.

**TABLE 1:** Participant sociodemographic characteristics.

Variable	*n*	%
**Gender**		
Men	9	64
Women	5	36
**Age (years)**		
22–35	6	43
36–59	8	57
**Condition**		
Diabetes	4	28
Hypertension	5	36
HIV	5	36

HIV, human immunodeficiency virus.

### Themes and subthemes

The study identified three major themes and eight sub-themes (Table 2).

**TABLE 2:** Themes and sub-themes that were identified during the study.

Themes	Sub-themes
Individual factors enhancing self-management	Understanding the chronic disease
	Attitudes and behaviours towards the chronic disease
	Self-motivation
	Disclosure of chronic disease status
Support enhancing self-management	Support from family and friends
	Support from healthcare workers
Barriers to self-management	Community influences
	Financial barriers

#### Theme 1: Individual factors enhancing self-management

Individual self-management refers to the cognitive, psychological and behavioural strategies used by people living with chronic illnesses to manage and support their health.

**Subtheme 1.1: Understanding the chronic disease:** Understanding of chronic disease encompasses knowledge of the nature of the chronic condition, knowledge of healthy lifestyle and behaviours and knowledge of treatment and the times that treatment should be taken.

Most of the participants demonstrated knowledge of the nature of the chronic disease that they are living with, for example, that it is a lifelong disease that requires daily treatment. One participant expressed that living with HIV is like living with other chronic diseases, which makes it easier for her to accept the condition and take care of herself. She further mentioned that she does not judge or feel sorry for herself:

‘HIV is like other diseases, just like high blood, just that it wants you to tell yourself that as much as you have the disease, you must take your treatment and follow all the instructions given at the clinic. You do not have to judge yourself and feel sorry for yourself. You must tell yourself that your condition is the same as that one with leg problem, hypertension patients and diabetes patients.’ (Participant 2, woman, 56 years old, HIV)

Other participants showed an understanding that some of the signs and symptoms of chronic disease can go undetected, but that it can be diagnosed and monitored by healthcare workers:

‘According to me, high blood is a silent killer; you cannot tell when someone has it, only healthcare workers can tell with the machine they use that you have high blood.’ (Participant 3, man, 30 years old, hypertension)

In addition to knowledge of the nature of chronic disease, some participants also knew what constitutes a healthy lifestyle and healthy behaviours. This included knowledge of a healthy diet, exercise and avoiding certain foods:

‘They have taught me about how to take care of my condition, like eating healthy, avoiding fats and salt, so it is much better when you know that you have it because you will be able to take care of yourself.’ (Participant 3, man, 30, hypertension)

‘High blood it is a struggle because it can kill you without noticing.’ (Participant 3, man, 30 years old, hypertension)

All the participants were taking treatment to manage their chronic disease. Most of the participants knew the treatment they were taking, but this knowledge was only related to the time the medication should be taken:

‘I don’t know the names of the medication that I am taking, but I take my medication. Once a day, I take three medications every day.’ (Participant 4, woman, 49 years old, hypertension)

‘I do not know the name of the medication, but I take one pill daily at 20:00 pm before I sleep.’ (Participant 6, man, 48 years old, HIV)

Some participants had a poor understanding of the condition they are living with. They had a very basic understanding that the disease is dangerous if medication is not taken:

‘I don’t really know much about HIV. I only know that it is dangerous, especially if you do not take your treatment after diagnosis.’ (Participant 6, man, 48 years old, HIV)

**Subtheme 1.2: Attitudes and behaviours towards the chronic disease:** A patient with chronic disease who believes that lifestyle modifications can improve their health and is willing to actively participate in their treatment has a positive attitude toward self-management. Beliefs or attitudes were identified as a key component of individual self-management. A person can have positive beliefs or negative beliefs. Many participants accepted their chronic disease for the sake of their family or children, or because of support received from family or friends. Others accepted the condition, realising that it is something that they cannot change:

‘I learned to accept what I cannot change and to do what I can to better myself and that you do not have to keep quiet, other people might know something or have gone through what you are going through in life.’ (Participant 14, man, 45 years old, diabetes)

Some participants living with HIV mentioned that HIV should be viewed like other diseases, so that it is easier for people to accept it. On the other hand, some participants mentioned that accepting living with a chronic condition was difficult. What made it difficult was the realisation that it is a chronic and lifelong condition:

‘Nothing has changed, just that it is sometimes difficult to accept that I am and will be HIV forever.’ (Participant 5, woman, 24 years old, HIV)

Most of the participants expressed positive beliefs regarding their chronic condition. This included positive beliefs about being able to live a normal life even when you have a chronic condition as well as the effectiveness of treatment:

‘What I believe about HIV is that take your treatment and advice that nurses give you, don’t focus on what people will say, you will be fine, and you have to tell yourself that since you are HIV, you need to take care of yourself.’ (Participant 2, woman, 37 years old, HIV)

‘I believe that high blood exists because if I don’t take my medication well, my body changes, my face becomes swollen. So, I believe for me to be okay, I must make sure that I take my medication.’ (Participant 4, woman, 49 years old, hypertension)

However, some participants also expressed negative beliefs. These included the fear of dying and leaving their children behind, or fears related to the potential negative effects of the disease itself:

‘But though sometimes you just feel like you are going to die because you see people dying every day.’ (Participant 2, woman, 37 years old, HIV)

‘I once heard that when you have diabetes you struggle sexually, I am not sure if it is true or not, my belief is that diabetes depends on how you take care of yourself.’ (Participant 10, man, 34 years old, diabetes).

Another key component of individual self-management was behaviours. Participants mentioned how they have been taking responsibility for their health by engaging in healthy behaviours and taking treatment. One participant mentioned that after the diagnosis, she volunteered to initiate antiretroviral treatment (ART), indicating her willingness to take responsibility:

‘I volunteered to begin with treatment because I was told that there is no need to start treatment because I was okay. I did not want the HIV to make me weak; hence I started the treatment.’ (Participant 2, woman, 37 years old, HIV)

Many of the participants demonstrated positive behaviour change following their diagnosis. This includes exercise, diet, reducing alcohol consumption or other lifestyle changes related to their condition, such as adherence and diet modification. These behaviour changes were advised by healthcare workers:

‘I eat normal food, vegetables, less sugar, and less cooking oil, pap and potatoes. At the clinic they said I should avoid sweet food, so I try every day to avoid it. I now eat vegetables, less sugar and less cooking oil.’ (Participant 10, man, 34 years old, diabetes)

‘The time I was not taking treatment, I was drinking alcohol a lot. But it was part of growing. I will be 60 years next year, changed of food, now I no longer eat food with chillies because ulcer and high blood will increase.’ (Participant 9, man, 59 years old, hypertension).

Although many participants mentioned positive behavioural changes, it was not easy for most of them to make the changes. Many participants mentioned that it was challenging to change their diet. Sometimes the challenges were related to financial constraints, food insecurity or the difficulty of adapting to a new diet:

‘Eating healthy is difficult; sometimes it is hard to make sure that all the healthy diet is available. Financially, it is a struggle, but I always try to make sure that I do have all the food that I must have just to be okay.’ (Participant 3, man, 30 years old, hypertension)

Participants also mentioned that it is challenging when their diet is different from other members of the family.

‘What changed is that food is not nice anymore, it does not taste the way it used to be because I do not use salt and cooking oil anymore and my family have to separate my food which is hard, but I have been trying my best to copy because diet also contributes to my health.’ (Participant 4, woman, 49 years old, hypertension)

Regarding medication self-management, many participants mentioned that although they take treatment every day, it is an activity that can be challenging, stressful and difficult to integrate into one’s daily routine. Participants thought medication adherence was ‘not easy’:

‘It is not easy to live with high blood and to take treatment, sometimes you forget to take your treatment sometimes you don’t.’ (Participant 3, man, 30 years old, hypertension)

Some participants mentioned that they did not engage in goal setting because it was not easy to achieve, or because of a lack of resources:

‘I have now adapted to my daily routine, I wake up, clean the house, cook, eat and take my medication, when I don’t feel lazy, I exercise but is not an everyday thing, is not easy to set goals because sometimes you don’t have enough resources like eating healthy, sometimes you don’t even have food.’ (Participant 4, woman, 49 years old, hypertension)

Controlling emotions was mentioned by patients as one of the challenges they experienced. Being told that they have a chronic disease can have a negative impact on their identity. Participants initially experienced several negative emotions, such as anger at the diagnosis of the chronic illness, but they were able to control their emotions on their own:

‘To be diagnosed with high blood was helpful for me because I had an ulcer, I had pain in my intestines. I wouldn’t know that I had an ulcer if it was not the high blood. Since I know I choose not to get angry and to be cool even though at first it was challenging because it was hard to accept that situation, but now I am okay.’ (Participant 9, man, 59 years old, hypertension)

Furthermore, participants mentioned that knowledge of their chronic condition assisted in managing their emotions as they had a better understanding of the disease and their symptoms:

‘I don’t remember very well, but what was happening is that I lost a lot of weight, and I was curious as to what was happening with my body, as much as it was painful, but I was grateful that I got to understand the cause of my weight loss.’ (Participant 6, man, 48 years old, HIV)

**Subtheme 1.3: Self-motivation:** Most of the participants were motivated to self-manage their chronic illness because they wanted to live long, stay healthy and be present to take care of their children:

‘I always think about my family; I have realised that if I continue looking after myself, I will live long enough until they are responsible and old enough to look after themselves.’ (Participant 4, woman, 49 years old, hypertension)

Some participants took their treatment for fear of negative consequences, such as disability or death. Not wanting to die was mentioned as a motive for medication adherence. Another motivating factor for one participant living with HIV was the prevention of vertical transmission:

‘Knowing that if I don’t take care of myself, I will die motivates me to take care of myself. I don’t want to die, especially now that I am pregnant, I want to make sure that my baby is not infected and that I live to take care of her.’ (Participant 7, 22 years old, woman, HIV)

Participants had experiences of family members who suffered poor health because of the negative consequences of chronic disease, which motivated them to take care of themselves:

‘My health and the thought of not achieving many goals in my life, like living long and being healthy. I don’t want to have a stroke. I saw how my grandmother passed on; I don’t want to see myself in the same situation.’ (Participant 3, man, 30 years old, hypertension)

**Subtheme 1.4: Disclosure of chronic disease status:** Some participants found disclosure to their families challenging, whereas others felt that it was easy to disclose the condition to people they live with. Participants mentioned that disclosure is important to them because they want their family to know what to do when they become sick one day, and so that they can provide support:

‘It is good because for me, my family members are traditional healers, I realised that if I don’t tell them, one day when I am sick, they will give me traditional treatment instead of taking me to hospital, so it was important.’ (Participant 2, woman, 37 years old, HIV).

‘I shared with my boyfriend and my mother so they can be able to support me.’ (Participant 11, woman, 33 years old, diabetes).

Many participants felt comfortable disclosing to family because they were not judgemental or because they felt that open and truthful communication reduces judgemental attitudes:

‘I told my mother, she told me to be strong and take my treatment, she encouraged me to do it for myself.’ (Participant 10, man, 34 years old, diabetes)

‘When you tell them the truth, they do not judge, because you were open to them from day one.’ (Participant 2, woman, 37 years old, HIV)

Although many participants disclosed to close family members, disclosure was still not easy. Some participants opted not to disclose to family members or partners as a result of confidentiality and trust. One participant mentioned that she only disclosed after her mother had accidentally found her hidden tablets and after she was assured by her mother that she would support her:

‘It took me time to tell my family, I did not tell my mother directly, she found the medication under the pillow and when we were in town, she just said that she was talking to a relative, and he mentioned that if he had a child living with HIV, he would support them, so it touched me, I decided to tell her about my status.’ (Participant 5, woman, 24, HIV)

Another participant mentioned that he did not disclose because he considered his chronic disease status confidential:

‘I won’t lie; I did not disclose to my friends. Remember that you cannot just share personal matters with people. Whenever my girlfriend sees me taking my medication, I always lie and say is for asthma.’ (Participant 3, man 30, hypertension)

#### Theme 2: Support enhancing self-management

Support for enhancing self-management was the second theme that was identified. This theme entails the support received from the family, friends and healthcare workers.

**Subtheme 2.1: Support from family and friends:** Several participants demonstrated the psychological support that they have been receiving from their families and friends. Managing a chronic condition can take a toll on a person’s mental health. It often involves dealing with ongoing symptoms, lifestyle adjustments and potential emotional challenges. Participants mentioned examples of their families providing emotional support:

‘I don’t think it was going to be easy for me without them; they have been helpful by comforting me when I am emotional.’ (Participant 9, man, 59 years old, hypertension)

Many participants regard their families and friends as their pillars in the management of the chronic diseases they are living with, from assisting with treatment and behaviour change to encouragement and being there for them all the time. Participants felt they were never alone:

‘My family has been supportive; they cook for me because I have to eat before taking the medication.’ (Participant 13, man, 55 years old, hypertension)

In the context of chronic diseases, family members must know the specific condition and its management. This knowledge helps in providing effective support, fostering understanding and creating a supportive environment for the individual with the chronic disease. Several participants mentioned that although their family members were supportive, they did not have in-depth knowledge about the type of condition they were living with:

‘They all know that I have high blood, unfortunately, they don’t have any idea about high blood, it is only the professional ones that know high blood.’ (Participation 8, man, 47 years old, hypertension)

Even though many family members had limited knowledge of the chronic disease, they were key to supporting participants with behaviour and lifestyle changes. Participants mentioned how their families encouraged them to adopt healthy behaviours such as exercising and eating healthy food. Some family members even changed their own behaviours or made it easier for participants by preparing healthy meals or joining them for exercise to encourage the participants. These actions provided positive motivation for continued behaviour change:

‘They cook and remind me to take treatment, they even exercise with me, it gives me power to see how they even join me so that I don’t quit.’ (Participant 9, man, 59 years old, hypertension)

‘Like I said, my mother reminds me to take my medication, she even reminds me to walk instead of taking a taxi sometimes.’ (Participant 10, man, 34 years old, diabetes)

**Subtheme 2.2: Support from healthcare workers:** The participants mentioned the support they received from the healthcare workers. The support was predominantly related to health education about healthy behaviours:

‘I was then advised by the nurses to try 1 litre of water; that’s when I became okay. The advice and health education from the healthcare workers are helpful.’ (Participant 2, woman, 56 years old, HIV)

While some participants praised healthcare workers for their support, others had different opinions and were unhappy with the attitudes and lack of professionalism of healthcare workers:

‘Some healthcare workers are nice, others are not. They will shout, give you attitude, it’s a lot.’ (Participant 13, man, 55 years old, hypertension)

‘I once had an argument with an admin clerk who kept passing me, other than that I have never had any challenges.’ (Participant 14, man, 45 years old, diabetes)

Participants acknowledged that certain health system processes have been put in place to make it easier for them to access treatment and care. Two of these systems mentioned were medication pick-up points and support groups or clubs. A pickup point model of care is an innovative approach to healthcare delivery that involves providing certain medical services or resources at designated pickup locations, rather than traditional healthcare facilities like hospitals or clinics:

‘So, when I started at the facility, they told me that if I can be able to take my medication well and make sure that my sugar level is normal, I will qualify to go collect my medication outside the clinic, where there are no lines. I made sure that my sugar level was normal, then I qualified, I chose as my pickup point, I go there every two months to collect my medication.’ (Participant 12, man, 32 years old, diabetes)

Support groups and youth clubs were mentioned by some participants as a solution to the overcrowding of the facilities:

‘So, we are given dates where we must go to the clinic; we meet as youth, the teachers (nurse and a counsellor) are able to educate us about HIV and how to live with HIV. Our medication is always prepacked when we get to the clinic. We just collect and go.’ (Participant 5, woman, 24 years old, HIV)

The healthcare system faces numerous challenges that impact its ability to provide accessible, affordable and high-quality care to individuals and populations. One of the challenges mentioned by participants includes long queues because of overcrowding of facilities:

‘My experience is bad; you wait in the queue for many hours until you are hungry.’ (Participant 10, man, 34 years old, diabetes)

‘The facility is always full, but because I arrive early, I don’t experience any challenges.’ (Participant 13, man, 55 years old, hypertension)

#### Theme 3: Barriers to self-management

Barriers to self-management is the third theme that was identified.

**Subtheme 3.1: Community influences:** Negative community influences that impeded self-management encompassed stigma, judgemental behaviour and lack of knowledge in the community:

‘In the community, there are people who are supportive of HIV regardless of whether they know your status or not, you can hear their opinions that they do not have a problem with people living with HIV, but there are also people who are against people who are living with HIV but all in all, I feel like at least the stigma of HIV is fading, people are becoming aware of the disease.’ (Participant 2, woman, 37 years old, HIV)

‘The community believe that people who are at risk of having high blood are old people, and if you tell them, you have high blood, they are always shocked and comment with silly comments like, you are going to die.’ (Participant 13, man, 55 years old, hypertension)

From the above quote, it is apparent that participants experienced people in the community in general as demonstrating stigmatising behaviours against people living with chronic diseases. It may be because of a lack of knowledge regarding the condition.

Some participants experienced stigma as being mostly directed towards people living with HIV, although it was improving:

‘I have never heard the community comment on hypertension; they mostly talk about HIV.’ (Participant 9, man, 59 years old, hypertension)

‘I feel like at least the stigma of HIV is fading; people are becoming aware of the disease.’ (Participant 2, woman, 37 years old, HIV)

**Subtheme 3.2: Financial barriers:** Participants generally lacked the financial resources to support positive behaviour change:

‘Not really, I try my level best to have everything I need for my health, and I always have my medication, though diet is a bit of a challenge because of finances.’ (Participant 14, man, 45 years old, diabetes)

‘The food that I eat is different to what I used to eat back then, and sometimes I don’t afford what I am supposed to eat.’ (Participant 10, man, 34 years old, diabetes)

## Discussion

The study aimed to explore the support that persons living with chronic disease require to self-manage their conditions in a semi-rural community. Three themes were identified: individual factors enhancing self-management, support enhancing self-management and barriers to self-management. Several individual factors enhanced self-management, for example, understanding of the disease, attitudes, self-motivation and disclosure. Several participants demonstrated limited understanding of their chronic disease, its causes, associated risk factors and effective management strategies.

Education is a critical component of chronic disease self-management, as highlighted in a study conducted in Limpopo, which showed that patients in a low-income rural community in Limpopo achieved improved blood pressure control when they received targeted self-management education.^4^ Equipping individuals with knowledge enhances their capacity to actively manage their health.^13^

Self-management is also influenced by attitudes and behaviours towards the chronic disease. Some participants in the present study felt hopeless or overwhelmed by their health status and struggled with acceptance, which in some cases influenced treatment adherence. These findings align with existing literature indicating that beliefs significantly affect treatment adherence, self-care behaviours and overall well-being.^14^ While many participants successfully adopted new behaviours such as engaging in physical activity, reducing alcohol consumption and following a balanced diet, others found it difficult to manage their emotions and make sustainable behavioural changes.^15^ Behavioural change is central to chronic disease self-management and requires the development of long-term habits, including medication adherence, lifestyle modifications and coping mechanisms.^15^ Although goal setting is a key strategy in managing diseases such as HIV, hypertension and diabetes, participants in this study often felt unable to set goals as a result of financial resource constraints.

Disclosure of one’s health status emerged as another complex aspect of self-management. Some participants were reluctant to disclose their condition because of stigma and fear of judgement, while others disclosed primarily to close family members. Research suggests that disclosure can play a pivotal role in enhancing self-management, as it often leads to greater emotional and practical support.^16^

In this study, participants who disclosed frequently received reminders to take medication and emotional support, while those who did not disclose reported limited assistance. This underscores the importance of supportive relationships in facilitating effective self-management. Making lifestyle adjustments is often essential to avoid complications and promote overall well-being in individuals living with chronic diseases. Healthcare providers, families and friends, therefore, have a vital role in offering guidance, nonjudgmental support and access to resources to help individuals navigate their health journeys.^1^

Participants in this study mostly received support from family and friends and healthcare workers. Family and friends often joined them in lifestyle changes such as exercise, assisted with appointments and daily routines, and reminded them to take their medication.

Healthcare workers supported participants, primarily through education. However, poor communication, which may be attributed to overcrowded facilities and limited consultation time, was a common concern. Poor communication and limited engagement with patients have been identified as a barrier to treatment adherence.^14^ While some participants acknowledged receiving health education, particularly about medication adherence, which they found beneficial, many expressed a need for more personalised attention and a collaborative, patient-centred approach to managing their disease. This highlights the importance of a comprehensive strategy involving both education and empathetic provider-patient relationships.^17^

Support groups emerged as another key resource, offering not only health education but also a sense of community and emotional reinforcement. Participants who attended these groups reported enhanced self-management skills and improved self-confidence, aligning with evidence that support groups significantly contribute to the well-being of individuals with chronic diseases. The support groups assisted patients in feeling less isolated, more confident, and more able to adhere to treatment guidelines.^18^ Additionally, participants highlighted the benefits of receiving medication through the external pick-up points of the CCMDD programme, noting that these sites offered greater convenience, shorter queues, flexible collection hours and reduced need for frequent clinic visits.^18^

Barriers to self-management included contextual factors such as community influences and financial barriers. Some participants continued to experience stigma, particularly related to HIV, which hindered their willingness to disclose their status and seek support. Encouragingly, some participants observed that HIV-related stigma is gradually diminishing as public awareness increases. Nonetheless, they felt that community understanding of other chronic diseases, such as hypertension and diabetes, remained limited, contributing to a lack of broader social support for individuals managing these diseases.^19^ Financial constraints emerged as a persistent barrier to effective self-management, with many participants reporting difficulties in affording transportation and healthy food.

These findings underscore the importance of accessible, person-centred systems of care that address self-management support across individual, interpersonal and contextual levels by integrating family involvement, community-based resources, and responsive health services to enable effective chronic disease self-management.

### Strengths and limitations

To the best of our knowledge, this is the first study to explore self-management support needs in a South African semi-rural context across various chronic diseases, thereby providing important contextual information that could assist in strategies to empower people to take care of their health in collaboration with their families and healthcare workers. However, the study has limitations, such as sample size constraints or participant diversity, which may have an impact on the generalisability of the findings. Furthermore, regional resource restrictions and cultural characteristics unique to Mpumalanga may limit the broader application of the findings to other situations.

### Recommendations

Key recommendations from this study include the development of tailored educational programmes to strengthen chronic disease self-management knowledge, including emotional management, symptom monitoring, medication adherence and lifestyle modification. Healthcare providers should collaborate with patients to set personalised and realistic self-management goals aligned with patients’ beliefs, interests and lifestyles.

The study also recommends strengthening healthcare provider training in areas such as motivational interviewing, goal setting and patient-centred counselling to better support individuals and families living with chronic conditions. Given time constraints within PHC facilities, educational pamphlets and regular follow-up counselling sessions should be prioritised to improve access to information and ongoing support.

In addition, there is a need to explore and expand digital self-management interventions, such as mobile applications and reminder systems, within the South African context to support chronic disease management. Community education initiatives led by the DoH and non-governmental organisations are also recommended to address stigma, misconceptions, and discrimination related to chronic diseases.

Finally, the development of resource guides outlining available financial support services, transport options, and community resources may assist persons living with chronic diseases in overcoming financial resource barriers to effective self-management.

## Conclusion

Knowing the support persons living with chronic diseases require for self-management is critical for improving health outcomes and quality of life and ensuring the general viability of healthcare systems. This study demonstrates that patients require comprehensive care that extends beyond healthcare and includes emotional, social and financial support. Effective self-management requires access to tailored education, flexible healthcare options and community support networks that allow people to take an active role in their health.

By addressing these diverse needs, healthcare providers can promote a more patient-centred approach that values each person’s particular challenges and strengths. Finally, providing self-management support to patients living with chronic disease has the potential to reduce complications, increase patient independence and improve long-term health outcomes.

## Appendix 2

### Participant characteristics

**Table:**

1. Age
2. Sex
3. The type of disease

The age, sex, the type of disease of the patients will be asked from the participants.

### 1. Introduction

Thank you for agreeing to participate in this study that aims to explore self-management support needs of persons living with chronic disease. Can I just confirm that you signed the informed consent document?

### 2. Objective 1: To explore understanding and beliefs

2.1 Tell me a bit more about yourself and the chronic illness you live with?

2.2 When were you diagnosed?

2.3 Tell me about the medication and/or other treatment that you are taking for the disease(s).

2.4 Tell me more about what you know or believe about your illness. (Probes: how do you feel about living with…? what does your family or community believe about this disease?)

2.5 Did you have to make any changes to the way you live since you have been diagnosed? Tell me more about that.

### 3. Objective 2: To explore self-regulation skills and abilities

3.1 Tell me more about what you do every day to take care of your health and chronic disease(s).

3.2 What do you find easy in taking care of your health and chronic illness?

3.3 What do you find difficult or challenging in taking care of your health and chronic illness?

### 4. Objective 3: To describe the resources

4.1 Tell me more about who supports you to take care of your health and chronic disease.

4.2 How is the support from family regarding your disease?

4.3 Describe your experiences with the health care system related to your diseases.

4.4 Describe your experiences with your health care providers?

4.5 Do you access any other resources that help you live with your chronic disease? Tell me about these.

4.6 How would you improve on the support and resources that are available to you?

4.7 Is there anything else that you would like to share about your experience of living with a chronic illness?
